# Reward frustration and withdrawal from work in health care—a cross-sectional study among health professionals

**DOI:** 10.3389/frhs.2025.1498073

**Published:** 2025-03-12

**Authors:** Oliver Hämmig

**Affiliations:** Epidemiology, Biostatistics and Prevention Institute (EBPI), University of Zurich, Zurich, Switzerland

**Keywords:** effort-reward imbalance, work stress, job performance, work engagement, work absenteeism, unpaid leave, job change, career ending

## Abstract

**Introduction:**

The health-related consequences of work stress are as broadly studied in the health care sector as they are elsewhere. However, behaviors such as underperforming at work, being less engaged at work, being habitually absent from work without good reason, intending to take unpaid leave, changing jobs or leaving the profession as consequences or correlates of stress and reward frustration at work are largely underresearched, particularly in Switzerland and in health care and across different health professions.

**Methods:**

Cross-sectional survey data collected from the workforces of six public hospitals and rehabilitation clinics in German-speaking Switzerland were used for this observational study. A total of 1,441 health care workers from various professions participated in the survey. The study focused on effort–reward imbalance (ERI) as a work stress measure and on six different withdrawal behaviors. Relative frequencies stratified by health professions for all study variables (exposure, confounders, and outcomes) and multiple-adjusted odds ratios as measures of association were calculated.

**Results:**

The findings revealed frequent work stress or rather widespread ERI among health professionals (49%). The results further revealed strong to very strong dose-response relationships between work stress levels and the chance or risk of withdrawal from work. Compared with the least stressed individuals, the most stressed individuals were at significantly increased risk for reduced job performance (aOR = 5.2), low work engagement (aOR = 4.4), increased work absenteeism (aOR = 2.2), and intentions to take unpaid leave (aOR = 3.1), to change the job (aOR = 35.0) or to leave the profession (aOR = 12.3).

**Conclusion:**

Highly prevalent reward frustration in Swiss health care needs to be reduced to overcome inner resignation and resistance and to prevent health professionals from withdrawing from work, and health care from high follow-up costs above and beyond mere absences from work or high turnover.

## Background

The health-related consequences of occupational stress have been broadly studied, predominantly in the health care or public sector. Numerous studies have shown that high workloads and, particularly, high work efforts (“costs”) coupled with comparably low rewards (“gains”) and the resulting reward frustration and “gratification crisis” on the job correlate with diverse poor health outcomes, disorders and diseases. Notably, the aforementioned “gratification crisis” is defined as work stress according to Johannes Siegrist (1) and his much-noticed stress concept and so-called effort–reward imbalance (ERI) model.

Many of these studies have been carried out among health care workers, particularly among nurses or physicians, and most of them have focused on and found associations between ERI and health outcomes, such as the following:

• Cardiovascular disease, particularly coronary heart disease (2–5),
• Burnout syndrome (6–10),
• Depression and other mental health problems (11–15),
• Musculoskeletal disorders (8, 16–18),
• Sleep disorders (16, 19–22), and
• Sick leave or sickness absence from work (13, 23–26).

However, before such serious health problems, particularly clinically relevant disorders and diseases, occur as a result of chronic work stress, people usually or preferably tend to cope with such a lack of reciprocity and frustration at work and bring their efforts and rewards at work into a self-perceived balance by simply avoiding long-lasting high-effort/low-reward situations or conditions at work or by actively reducing the effort put into work and/or increasing the rewards received from work. These efforts can be physical, temporal, emotional or motivational. In addition, rewards can be financial or nonmonetary and formal (wage increase, promotion, training, etc.) or informal (recognition of supervisors, appreciation of colleagues, etc.). A wide range of possible (and observed) coping strategies and work-related stress reactions exist, ranging from inner resignation or resistance (disengagement at work, minimizing one's effort, lateness, absenteeism) to withdrawal from work or working life (self-chosen reduction of one's employment level, temporary time-out and unpaid leave, intention to turnover, change of job or profession, early retirement and career ending).

However, in contrast to the abovementioned health-related consequences, such coping strategies or rather avoidance and withdrawal behaviors are much less studied as possible consequences or correlates of work stress and ERI, particularly in health care workers. Although few studies have investigated how ERI is associated with disengagement at work (27, 28) and with intentions to turnover or leave the (health) profession (7, 29–31), these behaviors or reactions to stress and frustration at work are largely unexplored in health care, health (service) research, and different health professions. This research gap is particularly true for health care professionals and hospital staff in Switzerland, where associations of ERI with more than just one individual withdrawal behavior and/or across different health professions have not been studied so far.

Consequently, the present study aimed to investigate the occurrence of some of these work-related avoidance strategies and withdrawal behaviors as a function of work stress, i.e., depending on the degree of ERI, in a large and diverse study population of health professionals employed in different public hospitals and rehabilitation clinics in German-speaking Switzerland.

The aforementioned avoidance strategies and withdrawal behaviors in the following are synonymously used umbrella terms to describe and summarize assumed or proven job stress responses, i.e., reactions to reward frustration and “gratification crises” at work. These strategies or behaviors are observed and/or expected to be highly prevalent, particularly in high-stress occupations and industries that suffer from high workloads, adverse and stressful working conditions and staff shortages, which further aggravate the problem of ERI and insufficiently rewarded high demands and efforts at work. Therefore, such behaviors are hypothesized to be most prevalent among heavily stressed health care workers, particularly among those most affected and stressed. These job withdrawal behaviors are further expected to be especially common among individuals with relatively high levels of job overcommitment. Even more, they might be primarily or at least partly attributed in truth to such job overcommitment as a personal trait or coping style rather than to work stress as a situational condition. Job overcommitment is known as the tendency to overreach or overspend oneself at work. This tendency was conceptualized by Siegrist and colleagues (32) as an excessive work-related commitment and an additional strong work stressor that is independent of conditions of combined high effort and low reward at work as the main cause of work stress.

In contrast, strong identification with or commitment to the workplace, the organization or employer can help individuals cope with or prevent work stress and frustration as a result of insufficiently rewarded high effort at work. Organizational identification or commitment has long been acknowledged and conceptualized as the individual's psychological, emotional or affective involvement in and attachment to an organization (33, 34), the employee's level of identification with the goals and values of the organization (35, 36) or a person's sense of belonging to the organization in which he or she works (37). Organizational commitment has long been proven to predict and prevent job withdrawal intentions or behaviors such as turnover or absenteeism and has also been found to be (positively) related to job performance or work productivity (39–41).

Usually, job stress is considered one of the causes or antecedents of (low or reduced) organizational commitment (42–44). In return, strong organizational commitment can reversely increase the willingness and dedication to make high efforts at work and therefore protect against reward frustration at work or, rather, prevent gratification crises and stress at work. No matter if it is an antecedent or a consequence of work stress, organizational commitment is expected to be closely related to a perceived ERI at work. Therefore it is additionally included in the present study as a potential confounding variable. In contrast to job overcommitment which is considered as an additional and independent risk factor for work stress, organizational commitment in this study is understood as a protective factor against work stress and withdrawal behaviors.

In sum, the present study addresses the following research questions:

• Do different health professionals (nurses, physicians, and therapists) show or prefer different work-related avoidance strategies and job withdrawal behaviors independent of their stress levels?
• Can these strategies and behaviors be observed with varying frequency or likelihood by levels of work stress among health professionals and hospital employees?
• Is there a positive dose‒response relationship between the level of work stress and the frequency and relative risk of being disengaged at work, being habitually absent from work due to demotivation (and not for health reasons), showing a job performance below average, intending to take unpaid leave or to quit the job or leave the profession?
• Is overcommitment to the job the expected additional work stressor and independent risk factor with regard to such undesired side effects and work-related outcomes and stress responses?
• Does an individual's strong commitment to the workplace and the organization buffer against work stress as presumed and therefore prevent proven work-related stress responses such as performing poorly at work (underperformance), being less involved, dedicated, motivated, concentrated and absorbed at work (disengagement), frequently not showing up for work (absenteeism), taking unpaid leave (unpaid leave), quitting the job (job change) or even leaving the profession (early career ending)?

**Figure 1:**
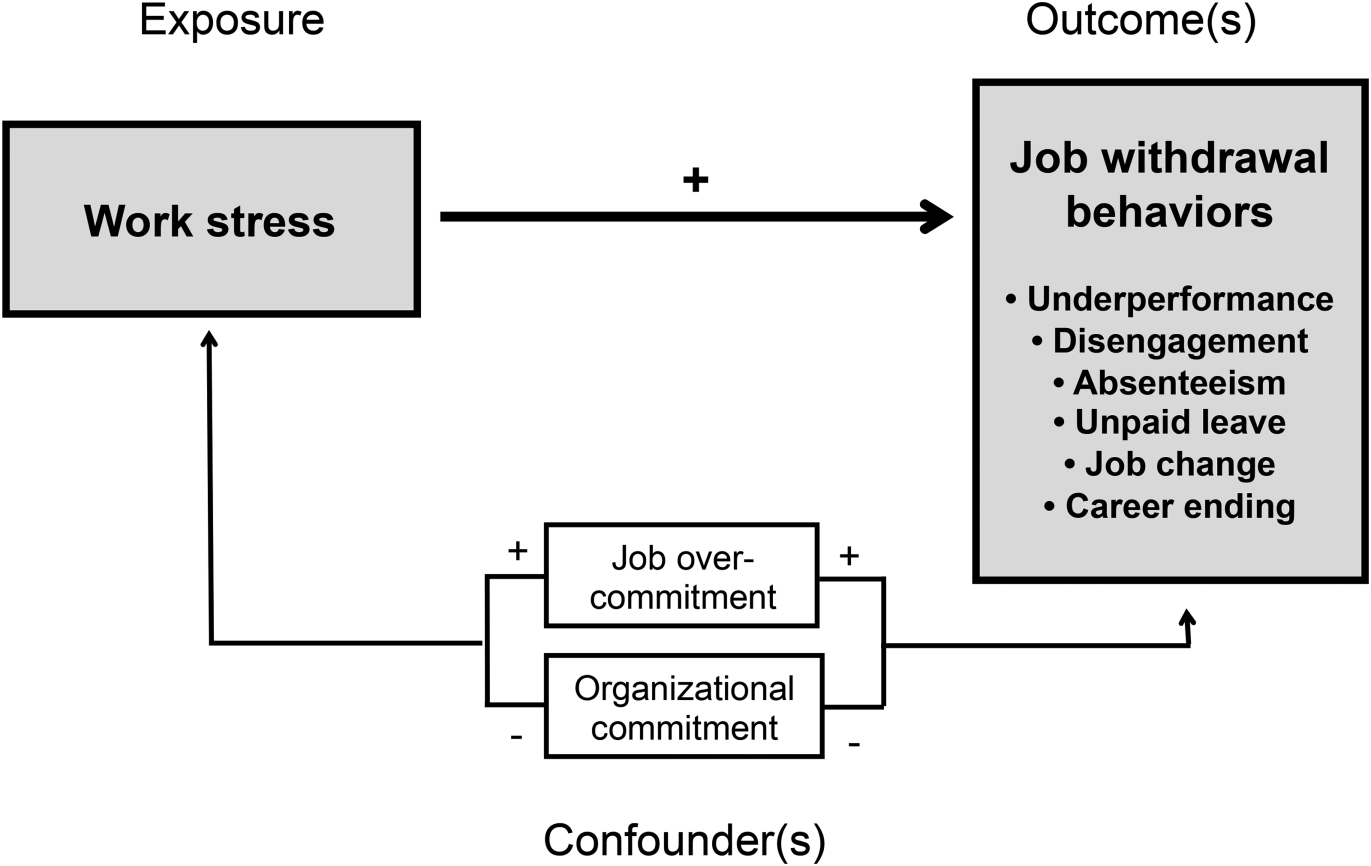
Theoretical model illustrating the causal paths and the hypothesized associations and intervening factors in the relationship between work stress and job withdrawal behaviors as work-related stress responses.

Figure 1 illustrates all assumed (direct and indirect) paths and associations, and the hypothesized confounders and stress outcomes as described in the research questions.

## Methods

### Data and study sample

The survey data used in this cross-sectional study were collected between summer 2015 and spring 2016 from the entire workforce of four public hospitals and two rehabilitation clinics in German-speaking Switzerland. Data were collected by a postal survey based on written questionnaires which were developed and provided by the University of Zurich and handed over by the participating hospitals and clinics to their employees together with pre-addressed envelopes and with prepaid postage and the university as the recipient.

The questionnaire entitled “Work and Health in the Hospital” consisted of exactly 100 single-item questions and multi-item scales with predefined answers. Pretests revealed that participants needed about half an hour on average to complete the questionnaire.

Approximately 4,450 employees were asked to participate voluntarily and anonymously in the full sample survey. After several reminders from employers, a total of 1,840 hospital employees completed and returned the questionnaire. The overall response or return rate of the survey was approximately 41%, whereas the return rates of the six participating health care institutions ranged from 36% to 49%. The return rate by health profession was also varying but could not be calculated since the exact amount of specific health professionals at the time of the data collection or during the provision and distribution of the questionnaires was not registered by the hospitals and clinics. Reports from individual hospitals and clinics revealed or rather suggested that there was presumably an oversampling of nurses whereas physicians most probably had a fairly low participation or response rate.

The study was restricted to a subsample of 1,441 health professionals, who were categorized into only four occupational groups or clusters (nurses, physicians, therapists, and other health professionals). 61.2% of the study sample were nurses (and midwives), 16.3% were physicians, an additional 11.0% were therapists and another 11.5% other health professionals.

More than 85% of the survey population (hospital employees) and almost 88% of the study sample (health professionals) were women, with a female proportion of more than 94% among caregivers and nurses (including midwives) and almost 64% among physicians (see Table 1). Almost three quarters (73%) of all participating health professionals had a higher educational degree with a high school diploma (general qualification for university entrance), a higher vocational school-certificate or a master degree of a university (of applied sciences).

**Table 1:** Socio-demographic characteristics of the entire study population (*N* = 1,441) and the four different groups of health professionals.

	Nurses	Physicians	Therapists	Other	All health professionals
	*n* = 882	*n* = 235	*n* = 158	*n* = 166	*N* = 1,441
Sex					
Women	94.3%	63.8%	84.8%	88.0%	87.5%
Men	5.7%	36.2%	15.2%	12.0%	12.5%
Age					
<25 years	9.0%	0.9%	3.2%	1.8%	6.2%
25–34 years	27.6%	32.2%	35.7%	34.3%	30.0%
35–44 years	22.9%	33.0%	28.0%	24.7%	25.3%
45–54 years	26.0%	21.5%	20.4%	25.9%	24.6%
55 + years	14.5%	12.4%	12.7%	13.3%	13.8%
Education (highest level achieved)					
Low (1–4)	5.3%	–	–	–	3.2%
Medium (5–6)	32.6%	1.3%	10.1%	22.0%	23.8%
High (7–10)	46.7%	0.9%	20.3%	33.5%	34.8%
Very high (11–12)	15.3%	97.8%	69.6%	44.5%	38.1%
Employer					
University hospital	44.2%	64.1%	23.6%	64.0%	47.4%
Cantonal hospital	12.8%	12.6%	14.6%	11.8%	12.9%
Regional hospital	33.7%	19.9%	25.5%	13.0%	28.2%
Rehabilitation clinic	9.3%	3.5%	36.3%	11.2%	11.5%

Table 1 also illustrates that the different health professions are somewhat unequally distributed over the four different types of health care institutions. Two thirds of all physicians (64%) and of the ‘other’ health professionals (64%) worked at a university hospital whereas only about one fourth of the therapists in the study sample (24%) were coming from a university hospital. At the same time more than one third of the participating therapists (36%), but only about one in 30 of the physicians (4%) were employed at a rehabilitation clinic.

## Measures

### Work stress, work stressor and work stress buffer

#### Work stress

Work stress as the main exposure variable was assessed with a well-established 16-item measure taken from the German version of the Effort–Reward Imbalance Questionnaire (ERIQ) of Siegrist et al. (45). A ratio was calculated by dividing the sum scores of the two subscales “effort” (6 items) and “reward” (10 items) and multiplying this figure by a correction or weighting factor (factor correcting for the difference in numbers of items of the two subscales): *effort/reward × correction factor (10/6)*. An ERI ratio of 1 or lower indicates a low or moderate level of work stress or, in other words, a sufficiently rewarded work effort. An ERI ratio above 1 is considered an imbalance between effort and reward at work or an underrewarded work effort and therefore a so-called gratification crisis or reward frustration, i.e., a high (ERI ratio >1.0 ≤1.5) or very high (ERI ratio >1.5) level of work stress.

#### Overcommitment to the job

Job overcommitment, conceptualized as a personal characteristic and coping pattern or style and characterized by an excessive work-related commitment and a high need for approval, was considered to be an additional work stressor and measured by a 6-item scale (OC-6) and the short version (32) of an originally 29-item scale (1). The following six questions with response options ranging from “strongly disagree” (score of 0) to “strongly agree” (3) were included:

• I am easily overwhelmed by time pressures at work.
• As soon as I wake up in the morning, I start thinking about work problems.
• When I arrive home, I can easily relax and switch-off from work.
• People close to me say I sacrifice too much for my job.
• Work rarely lets me go; it is still on my mind when I go to bed.
• If I postpone something that I was supposed to do today, I will have trouble sleeping at night.

A sum score was calculated, and the scale was then binary coded (dummy variable): A score of 11 to 18 was categorized as a comparably high level of over­commitment, and a score of 10 or below was categorized as a low or nonexistent overcommitment.

#### Commitment to the company

Organizational commitment or loyalty to an employer is defined as the extent or degree of an employee's psychological attachment to and identification with the organization or employer. The commitment to the workplace and employer as a potential buffer against job stress and a protector against withdrawal from work was measured by a 4-item subscale (CW-4) of the same name and developed by Kristensen and colleagues (46). This subscale was taken from the German standard version of the Copenhagen Psychosocial Questionnaire (COPSOQ) and included the following four items with five response options each, ranging from a “very small extent” (score 0) and a “small extent” (1) over “somewhat” (2) to a “large extent” (3) and a “very large extent” (4).

• Are you proud of being part of this organization?
• Do you enjoy telling others about your place of work?
• Do you feel that the problems at your place of work are yours, too?
• Do you feel that your place of work is of great personal importance to you?

A total score was calculated by summing the scores of each item. A total score of 12 or higher on a sum scale from 0 to 16 was categorized as strong commitment to the workplace.

### Job withdrawal behaviors

#### Job performance

Job performance was measured by a self-rated assessment of one's actual job performance compared with the best performance ever achieved in the past. A score of 5 or below on a scale from 0 (worst) to 10 (best) was considered as a relatively low or poor performance or an underperformance at work.

#### Work engagement

Work engagement was assessed by the short and German version of the Utrecht Work Engagement Scale of Schaufeli and Bakker (47) with a reduced set of 9 items (UWES-9) with the following response options about the frequency of feeling this way: never (score 0), almost never (1), from time to time (2), regularly (3), often (4), very often (5), and always (6). The following 9 items or statements were included.

• At my work, I feel bursting with energy.
• At my job, I feel strong and vigorous.
• I am enthusiastic about my job.
• My job inspires me.
• When I get up in the morning, I feel like going to work.
• I feel happy when I am working intensely.
• I am proud of the work that I do.
• I am immersed in my work.
• I get carried away when I'm working.

A total score of 22 or less on the 9-item scale with a maximum sum score of 54, indicating the highest level of work engagement, was considered low work engagement, i.e., being or becoming disengaged at work.

#### Absenteeism

Absenteeism refers to being habitually absent from work for motivational and not health reasons. Absenteeism in this study, particularly in the underlying survey, was assessed more as a work attitude rather than a behavior and by fully or somewhat agreeing on at least two of the following three statements, each with four response categories: fully agree (score 2), rather agree (1), rather disagree (0), and completely disagree (0):

• Some of the absences from work can be explained by adverse working conditions.
• Absences from work can often be attributed to low job satisfaction or a lack of motivation.
• It is hardly noticed or does not matter in a large company if someone is absent from work or taking sick leave for one or two days.

A sum score of three or more on the 3-item scale from 0 to 6 indicates full or partial agreement on at least two of the three items and therefore a rather positive attitude or even a certain tendency or predisposition toward absenteeism.

#### Unpaid leave

This variable was assessed by simply asking what respondents think about taking unpaid leave or rather about having the chance or option of going on unpaid (long-term) vacation (with response options of “already having it”, “not interested”, “not possible in my job”, or “wish to have”). Not yet having this option and/or not having the possibility or not being interested in it but, rather, wishing to have it was considered taking or wishing to take unpaid leave.

#### Job change

The intention of a change of job was measured by the following question: Have you ever seriously considered quitting and changing jobs since joining the company? The two response options “Yes, but no longer current” and “No, never considered” were categorized as not intending to change the job. The third possible answer “Yes, and since then nothing has changed” was categorized as resigning or intending to resign from the job.

#### Early career ending

The intention to give up the profession was measured by directly asking the survey participants how often they were thinking of leaving the profession over the past twelve months, with response options ranging from “never” to “daily”. Answering “several times per month”, “several times per week” or “daily” was considered as being about to leave the profession or as having frequent thoughts of prematurely ending one's own career.

### Control variables

Sex, age and education were used as control variables as it is well known and has been shown repeatedly in psychological stress and clinical health research that coping strategies differ with gender and age (48, 49).

### Analyses

To answer the research questions, descriptive statistics or, more precisely, frequency distributions of all the study variables and for different health professions separately (nurses, physicians, therapists, others) were calculated first. Then, bivariate correlations between all relevant study variables were calculated in order to test the relationships shown and implicitly hypothesized in the theoretical path model (Figure 1). Subsequently, crosstabulations were carried out, and prevalence rates (relative frequencies) of all six assumed and studied job withdrawal behaviors were calculated by different levels of work stress or degrees of ERI. Finally, multiple logistic regression analyses were performed, and adjusted odds ratios as proxies for the relative risks were calculated for increased levels of work stress or ERI in comparison with the reference group of those study (and survey) participants who did not show an ERI at all (low/moderate work stress). Adjustments were made for different control variables (sex, age, and education), and calculations were performed with and without consideration of potential confounders, such as personal overcommitment to the job and organizational commitment to the workplace (basic and extended model).

## Results

Some health professionals had significantly greater proportions of stressed individuals with (very) high degrees of ERI when compared to others: 54% of the nurses and ‘only’ 38% of the therapists showed increased levels of work stress and ERI ratios above 1, indicating an ERI or gratification crisis at work (see Table 2). Physicians and all “other” health professionals were between those of nurses and therapists with proportions of 43% or 44%, respectively of highly stressed individuals. However, almost half of the surveyed and studied health professionals in total felt underrewarded and frustrated at work and thus were (highly) stressed.

**Table 2:** Relative frequencies of all relevant study variables for the entire study population (*N* = 1,441) and the four different groups of health professionals.

	Nurses	Physicians	Therapists	Other	All health professionals
	*n* = 882	*n* = 235	*n* = 158	*n* = 166	*N* = 1,441
Work stress or effort-reward imbalance (ERI ratio)					
Low/moderate (≤1.0)	46.4%	57.0%	61.7%	56.1%	50.9%
High (>1.0–1.5)	44.9%	36.9%	30.2%	34.4%	40.7%
Very high (>1.5)	8.7%	6.1%	8.1%	9.6%	8.3%
Job overcommitment					
No/low (0–5)	20.1%	7.5%	13.0%	15.2%	16.7%
Moderate (6–10)	68.0%	64.9%	72.7%	67.1%	67.9%
High (11–18)	11.9%	27.6%	14.3%	17.7%	15.4%
Organizational commitment					
Weak (0–6)	17.2%	15.6%	9.0%	10.9%	15.3%
Moderate (7–11)	70.0%	61.5%	72.7%	60.6%	68.2%
Strong (12–16)	12.8%	22.9%	14.3%	28.5%	16.5%
Job performance					
Low (0–5)	7.1%	6.8%	7.0%	5.5%	6.8%
Moderate (6–7)	35.7%	29.5%	37.6%	33.3%	34.6%
High (8–10)	57.2%	63.7%	55.4%	61.2%	58.5%
Work engagement					
Low (0–22)	9.6%	11.6%	8.4%	15.1%	10.4%
Moderate (23–38)	59.9%	57.8%	63.9%	50.3%	58.9%
High (39–54)	30.6%	30.7%	27.7%	34.6%	30.7%
Attitude towards absenteeism					
Rather negative (0)	46.5%	51.6%	62.5%	54.7%	50.0%
Indifferent (1–2)	44.0%	39.5%	31.6%	37.7%	41.2%
Rather positive (3–6)	9.5%	8.8%	5.9%	7.5%	8.8%
Attitude towards unpaid leave					
Have I already (1)	29.8%	19.4%	33.5%	27.8%	28.3%
Not possible (2)/not interested (3)	30.1%	23.3%	23.3%	26.5%	27.8%
Wish to have (4)	40.1%	57.3%	43.2%	45.7%	43.9%
Intention to change the job					
Yes, and nothing has changed since then (1)	16.6%	12.6%	17.2%	18.9%	16.3%
Yes, but no longer current (2)	47.4%	35.9%	38.2%	43.9%	44.1%
No, never considered (3)	36.0%	51.5%	44.6%	37.2%	39.6%
Thoughts of leaving the profession					
Never (0)	47.0%	54.7%	50.6%	53.7%	49.4%
Several times per year (1)	34.4%	31.0%	35.4%	33.5%	33.9%
Several times per month (2) or week (3), daily (4)	18.5%	14.2%	13.9%	12.8%	16.7%

The frequency distributions shown in Table 2 further revealed that a strong commitment to the workplace was most common among physicians (23%) and “other” health professionals (29%) and was less common among nurses (13%) and therapists (14%). Similarly, high overcommitment to the job was found to be far more common among physicians (28%), than among nurses (12%).

Differences between the health professions regarding the six studied outcome variables or withdrawal behaviors (low work productivity, increased absenteeism, low work engagement, taking unpaid and long-term leave, resigning from one's job, and leaving one's profession) were less pronounced, with one exception: 57% of the physicians compared with “only” 40% of the nurses wished to go on unpaid vacation (see Table 2).

In total, almost half (49%) of all the studied health professionals experienced an ERI or gratification crisis and hence were highly stressed. This proportion varied significantly among health professionals and was highest among nurses (54%) and lowest among therapists (38%).

The correlation coefficients shown in the correlation matrix of Table 3 basically confirmed all assumed negative and positive associations illustrated in Figure 1. Work stress is positively correlated with absenteeism and with intentions to take unpaid leave, to quit the job or to leave the profession. Moreover, work stress is negatively correlated with job performance and work engagement. In addition, the intervening variables and potential confounders, organizational commitment and job overcommitment, are both inversely correlated with work stress as the exposure variable and all six outcome variables, as expected.

**Table 3:** Simple correlation matrix of all relevant study variables (scores, mean values, standard deviations, and Pearson's correlation coefficients r).

		Score	M	SD	1	2	3	4	5	6	7	8	9
1	Work stress (ERI ratio)	0.3–3.3	1.03	0.32	–								
2	Job overcommitment	0–18	7.86	2.67	.46***	–							
3	Organizational commitment	0–16	9.13	2.62	−.24***	.05*	–						
4	Job performance	0–10	7.63	1.32	−.15***	−.13***	.25***	–					
5	Work engagement	0–54	34.0	8.82	−.26***	−.17***	.48***	.44***	–				
6	Absenteeism	0–6	0.91	1.13	.20***	.07*	−.12***	n.s.	−.08**	–			
7	Desire for unpaid leave (dummy)	0/1	0.44	0.50	.19***	.14***	−.07*	−.08**	−.14 ***	n.s.	–		
8	Intention to change the job (dummy)	0/1	0.16	0.37	.45***	.25***	−.21***	−.16***	−.27***	.12***	.10***	–	
9	Thoughts of leaving the profession	0–4	0.71	0.89	.43***	.30***	−.31***	−.29***	−.42***	.13***	.14***	.47***	–

n.s., not significant (*p* > .05).

* *p* ≤ .05.

** *p* < .01.

*** *p* < .001.

A clear and partly very strong positive dose‒response relationship between the level of work stress and the prevalence of different job withdrawal behaviors was consistently found across all of these behaviors. A comparably low self-rated work productivity or job performance increased significantly with higher levels of work stress (see Table 4), starting from 4% at the lowest stress level (low or moderate degree of ERI), through 8% at the medium stress level (high degree of ERI) and finally up to 18% at the highest stress level (very high degree of ERI). The same applies to absenteeism which was measured by the reported attitude of health professionals that one's (own) absence from work for a few days is essentially not a problem and is due to adverse working conditions and to low satisfaction or a lack of motivation at work. A higher work stress level was associated with an increased prevalence of this attitude among health professionals, ranging from 7% to 16% (see Table 4). Low work engagement becomes significantly more common when the work stress level increases (see Table 4). Only 7% of the reference group (low or moderate ERI) but 11% of the highly stressed group and 27% of the very highly stressed group are less engaged at work.

**Table 4:** Multiple adjusted effects of work stress on job withdrawal behaviors among health professionals in Switzerland (*N* = 1,441).

		Underperformance on the job^2^			Disengagement at work^3^			Absenteeism^4^		
		%	aOR^1^	95% CI	%	aOR^1^	95% CI	%	aOR^1^	95% CI
**Total study population**		**6.8**			**10.4**			**8.8**		
Basic Model (Step 1)	Work stress (ERI ratio)									
	• Low/moderate (≤1.0)	3.7	1		7.0	1		6.6	1	
	• High (>1.0–1.5)	8.3	2.35***	1.42–3.90	11.3	1.75**	1.16–2.63	10.2	1.60*	1.04–2.46
	• Very high (>1.5)	18.0	5.52***	2.91–10.46	26.9	4.90***	2.88–8.32	15.9	2.57**	1.37–4.80
	No. cases in model		1,302			1,278			1,263	
Extended Model (Step 2)	Work stress (ERI ratio)									
	• Low/moderate (≤1.0)	3.7	1		7.0	1		6.6	1	
	• High (>1.0–1.5)	8.3	2.24**	1.33–3.78	11.3	1.66*	1.09–2.52	10.2	1.47	0.95–2.28
	• Very high (>1.5)	18.0	5.21***	2.64–10.26	26.9	4.36***	2.46–7.73	15.9	2.22*	1.15–4.30
	Job overcommitment									
	• Low/moderate (0–10)	6.2	1		9.9	1		8.1	1	
	• High (11–18)	9.7	1.06	0.59–1.92	13.1	0.99	0.59–1.66	12.1	1.32	0.77–2.26
	Organizational commitment									
	• Weak (0–11)	7.4	1		12.0	1		9.3	1	
	• Strong (12–16)	3.4	0.49	0.22–1.09	1.7	0.11***	0.03–0.35	6.3	0.73	0.40–1.32
	No. cases in model		1,271			1,250			1,231	
		Going on unpaid leave^5^			Resigning from job^6^			Ending the career^7^		
		%	aOR^1^	95% CI	%	aOR^1^	95% CI	%	aOR^1^	95% CI
**Total study population**		**43.5**			**16.3**			**16.7**		
Basic Model (Step 1)	Work stress (ERI ratio)									
	• Low/moderate (≤1.0)	36.9	1		4.4	1		7.3	1	
	• High (>1.0–1.5)	46.8	1.59***	1.25–2.03	21.3	6.04***	3.96–9.22	20.8	3.64***	2.52–5.25
	• Very high (>1.5)	66.7	3.42***	2.21–5.29	64.0	38.73***	22.58–66.44	53.2	17.47***	10.68–28.57
	No. cases in model		1,283			1,298			1,300	
Extended Model (Step 2)	Work stress (ERI ratio)									
	• Low/moderate (≤1.0)	36.9	1		4.4	1		7.3	1	
	• High (>1.0–1.5)	46.8	1.58***	1.23–2.03	21.3	5.57***	3.72–8.95	20.8	2.97***	2.03–4.34
	• Very high (>1.5)	66.7	3.09***	1.97–4.86	64.0	34.98***	18.77–57.95	53.2	12.32***	7.36–20.62
	Job overcommitment									
	• Low/moderate (0–10)	41.6	1		13.3	1		13.2	1	
	• High (11–18)	56.9	1.34	0.95–1.88	31.6	1.66*	1.10–2.52	34.7	2.62***	1.75–3.92
	Organizational commitment									
	• Weak (0–11)	44.0	1		17.7	1		18.9	1	
	• Strong (12–16)	42.3	0.88	0.64–1.21	8.2	0.52*	0.29–0.90	5.6	0.26***	0.14–0.50
	No. cases in model		1,250			1,265			1,266	

^1^ Odds ratios adjusted for control variables (sex, age, eduction); aOR = 1: reference category (comparison group), aOR with no *: *p* > .05 (not significant), aOR with *: *p* ≤ .05, aOR with **: *p* < .01, aOR with ***: *p* < .001.

^2^ Currently performing below average on the job or showing a job performance far below the best one ever achieved (score between 0 and 5 on a single-item scale from 0 ‘lowest’ to 10 ‘highest’).

^3^ Having rather low levels of energy, vigor, enthusiasm, inspiration, pleasure, happiness, pride, fulfillment and absorption at work (scoring 22 or below on a 9-item scale with a sum score from 0 to 54).

^4^ Having a positive attitude towards absenteeism, i.e., generally attributing absence from work to adverse working conditions, low job satisfaction, a lack of motivation and/or insignificance (scoring 3 or more on a 3-item scale with a sum score between 0 and 6).

^5^ Measured by wishing to have the chance or option to go on unpaid (long-term) vacation (sabbatical leave).

^6^ Measured approximately by the intention to resign from the job or more precisely by actually and seriously considering to quit or change the job.

^7^ Measured by frequently thinking about leaving the profession over the past 12 months (from several times per month to daily).

Unlike absenteeism, low job performance or work engagement, which are rarely observed, the desire for unpaid leave is widespread among health professionals. More than two-fifths of all the studied health professionals wish to have unpaid leave and even long-term vacation. While “only” 37% of the unstressed or least stressed health care workers wished to take unpaid leave, already 47% of the highly stressed and even 67% of the most stressed health professionals wished to go on unpaid leave and long-term vacation. The strongest dose‒response relationship with work stress, however, was found for intentions to quit or resign from the job and/or to leave the profession (see Table 4). While only 4% or 7% of the health professionals who do not reveal an ERI (low or moderate work stress) consider quitting or changing jobs or leaving the profession, 21% of the highly stressed health professionals and even 64% or 53% of the very highly stressed health professionals do so.

When odds ratios as measures or proxies of the relative risk were calculated and additionally adjusted for control variables, such as sex, age and education, odds ratios increased significantly and substantially with increasing stress levels (see Table 4). Compared with the reference group of health professionals with no or low to moderate stress at work, the risk or chance for the studied six job withdrawal behaviors was increased by a factor or odds ratio between 1.6 and 6.0 for the highly stressed health professionals. For the most or very highly stressed health professionals, this factor even was between 2.6 and 38.7. The strong associations and steep gradients with higher stress levels remained stable and only insignificantly decreased when the two confounders (or mediators) were included in the analyses (see extended regression models in Table 4).

## Discussion

Stressful jobs and various withdrawal behaviors at work are serious problems in the notoriously overloaded and understaffed health care system in Switzerland, as in other countries. Highly stressed (health care) workers basically have two choices to cope with such work stress. They can reduce stress and reward frustration at work either by avoiding high-effort/low-reward situations at work and reducing effort put into work or by inwardly resigning or permanently or temporarily withdrawing from work or working life. Such avoidance strategies and withdrawal behaviors have hardly been researched before as potential reactions to work stress, particularly not in this variety and combination and not in the health care setting (of Switzerland).

This study therefore not focused on health-related consequences of work stress in a narrow sense but rather on such avoidance strategies and withdrawal behaviors as potential stress responses or “coping strategies”, and additionally considered (a) overcommitment to the job as a personal trait and a proven trigger for work stress (in addition to high-effort/low-reward situations at work) and (b) organizational commitment as a recognized protective, stress-buffering factor.

The mentioned and postulated job withdrawal behaviors were as follows:

1. working with reduced performance and productivity,
2. being regularly and habitually absent from work without good reason or for reasons other than health;
3. being less involved, committed and engaged at work;
4. taking unpaid and temporary leave and going on long-term vacation,
5. resigning from one's job or
6. leaving one's profession.

These withdrawal behaviors, which can be considered or must be viewed as indicators of psychological or social adjustment to dissatisfying, frustrating and/or stressful working conditions, were not directly assessed and studied in this examination and the underlying survey, as they cannot be observed or easily and directly queried among the actual workforce. Instead of such (observed) behaviors, self-reports, attitudes or intentions were assessed and could have been studied accordingly. The indicators or proxies used for these withdrawal behaviors were the following:

1. performing relatively low in one's job compared to the past
2. showing sympathy for and not overestimating absences from work (of others) for reasons of dissatisfaction and demotivation
3. being unmotivated and uninspired at work and not being enthusiastic about the job or absorbed at work
4. wishing to have the chance or option to go on unpaid (long-term) leave
5. seriously considering a job change
6. frequently thinking about giving up the profession or career

The study results were not surprising, but the identified associations were surprisingly consistent and strong, even after adjusting for possible confounders and the usual control variables. Job overcommitment (stressor or risk factor) and organizational commitment (resource or protective factor) were found to be strong predictors or correlates of work stress and the outcomes under study but were not found to be substantial confounders of the relationships between work stress in the form of reward frustration and job withdrawal behaviors.

Comparably large proportions of the surveyed 1,441 health professionals, employees of public hospitals and rehabilitation clinics in German-speaking Switzerland, experienced an ERI or “gratification crisis” at work and, thus, felt frustrated and stressed due to an insufficiently rewarded although paid job. In total, almost 50% of the respondents were found to be (very) highly stressed at work. In addition, all of the studied job withdrawal behaviors are consistently accompanied by an ERI at a rate that far exceeds the average. Because strong to very strong associations and clear dose‒response relationships were consistently found, work stress, as measured by the ERI ratio, can be assumed to be a true cause and not just a correlate of job withdrawal behaviors. Not even the two potential confounders that were considered and included in the multiple regression analyses did substantially reduce these associations.

The results of this study revealed that work stress among health professionals, as expected and measured by the ERI model of Siegrist, is associated with a more than twofold elevated chance of being absent from work for motivational and not health reasons and with a more than threefold risk of taking unpaid leave and long-term vacation or at least wishing to do so. Analyses further revealed that the risks or odds of performing comparatively low and engaging relatively little at work are strongly growing with increasing levels of work stress up to a factor of 5 compared with those who are not or only slightly or moderately stressed and do not show any ERI. By far, the highest odds ratios associated with an ERI or rather an ERI ratio above 1.5 have been detected with respect to intentions to quit and change jobs (35-fold increased risk) or to leave the profession (12-fold increased risk).

All associations between levels of work stress or ERI and relative frequencies or risks of job withdrawal behaviors were not found to be substantially confounded (or mediated) by the two potential intervening variables of job overcommitment and organizational commitment.

Although no other study has focused on such a variety of withdrawal behaviors or intentions as possible consequences of an ERI at work and in health care, the results reported herein are largely in line with those of previous studies that examined individual aspects of the relationships investigated in this study. Leineweber et al. (29), in their 4-year longitudinal study from Sweden of a large cohort of working people, also found associations between ERI and employees’ turnover intentions. An earlier Belgian longitudinal study of 1,531 nurses analyzed the impact of ERI on the intent to leave the current organization or nursing profession (31). The prospective study, which was based on the Belgian subsample of the Nurses Early Exit (NEXT) study, showed that an imbalance between high efforts and low rewards significantly increases the risk of intentions to leave the organization (OR = 5.0) and the profession (OR = 1.8). Another prospective study by Li et al. (30), which was also based on data from the European longitudinal NEXT study, revealed that a high ERI mostly predicts the intention to leave the nursing profession for most of the seven studied countries (Belgium, Germany, France, Italy, the Netherlands, Poland, and Slovakia). However, associations in both longitudinal studies were not as strong as those in the present cross-sectional study, and a high level of overcommitment not turned out to be a significant additional predictor (31) or if at all in countries such as Germany, Italy or Poland (30).

A previous study also revealed that ERI is a strong predictor of thoughts of leaving the profession and an important mediator between different work demands or stressors and such thoughts (7). Although this study was based on the same data and not focused solely on health professionals but also on an extended sample of hospital employees, it did not consider potential confounders such as job overcommitment or organizational commitment. Another recently published study examined job stress and general stress as two different stress models and measures and their interplay with job resources in the prediction of job burnout and its antipode work engagement (27).

These two earlier Swiss studies have attempted to explain just one individual stress response or reaction (intention to leave the profession or disengagement at work) and have not focused on job stress and reward frustration or ERI as the sole determining factor. Instead, these studies considered additional stressors and stress measures, such as general stress or work‒life imbalance (7, 27). Therefore, these findings are not completely comparable with the present study's results.

In contrast, the present study does not consider the private life of its participants and instead solely focuses on job stress or ERI in working life. Moreover, the present study significantly extends the aforementioned two previous studies by examining a total of six and mostly unexplored potential stress responses or withdrawal behaviors among health professionals and by considering potential confounders, such as job overcommitment and organizational commitment, which might enhance or mitigate such stress responses or withdrawal behaviors.

In summary, the results of the present study partly support the findings of the few previously published studies, which were either restricted to specific health professions (nursing profession) or extended to the entire working population or were limited mostly to individual forms of withdrawal from work (intentions to leave the organization or profession). The associations found, particularly between ERI and intentions to leave the organization or profession, seem to be extraordinarily strong in the present study, much stronger than in other and earlier studies. Studies that have investigated other additional or various withdrawal behaviors simultaneously and specifically in the health care setting and among or across different health professions simply do not exist in the literature.

### Limitations

Study findings strictly speaking cannot be generalized neither to the health professions in general nor to the entire workforce in the hospital setting or even health care system because the survey participants and study sample do not completely represent the totality of health professionals as the participating hospitals and clinics were not systematically or even randomly selected from all public hospitals and clinics in German-speaking Switzerland. Self-selection of employers or health care institutions might lead to a systematic bias of the study results as only those hospitals and clinics were possibly willing to participate which have or anticipate comparably good working conditions or relatively satisfied employees. If ever such a selection bias would have been occurred here, work stress and stress responses or more specifically withdrawal behaviors might have been underestimated in their frequency or prevalence. Their association and not their relative frequency however was in the interest of the study and would presumably not have been systematically biased as a result of a lacking representativity of the study sample and a limited generalizability of the spread of such observed phenomena like work stress or job withdrawal behaviors.

Besides this, selection bias can also not be completely excluded in this study insofar as the participation or return rate of the questionnaire-based survey among hospital employees in total was “only” 41%, and self-selection could have occurred for reasons that may be systematically associated with the outcomes under study. For example, the most frustrated and stressed employees may have frequently refused their participation or may have overreported their true intentions to quit the job or similar intentions. However, the return rate of over 40% was not particularly low compared with that of similar surveys—rather, the opposite was true. There was no indication of systematic self-exclusion of the particularly frustrated and stressed individuals from the survey. Moreover, the survey was not introduced as a usual employee survey conducted or initiated by the company's management on its own interest and/or for reasons of profit-making or legitimacy and with a special focus and interest on job satisfaction and operational output parameters. Instead, the survey was introduced as a completely voluntary and anonymous data collection that was initiated and will be analyzed and interpreted by an independent scientific institution with an unselfish research interest in the working conditions and the health and well-being of hospital employees and health care workers.

Some of the measures used in this study are well-established whereas some others are just simple single-item questions used in the Swiss Health Survey and/or other population-based surveys in Switzerland. The well-established and validated instruments are the following: the 6-item and the 10-item ERI subscales on effort and reward at work and the ERI ratio calculated from them, the 9-item work engagement scale (UWES-9), the 6-item overcommitment scale (OC-6) and the 4-item commitment to the workplace scale (CW-4). Their validity and reliability is beyond question. Three other measures are unproblematic single-item questions which are directly pointing on the measured subject (intentions to go on unpaid leave, to change the job and to leave the profession).

Only in the case of job performance and absenteeism the construct validity of the used measures is possibly somewhat limited. Because they are delicate topics with a tendency to give socially desirable answers, and because established and validated measures for them do not exist. The self-assessment of the job performance might not be best to measure one's own and true job performance. And positive attitudes or lacking negative attitudes towards absenteeism of others and in general are presumably not exactly the same than being oneself regularly absent from work for motivational and not health reasons. Therefore, possible systematic biases could have occurred but most likely would have lead to an underestimation of the “true” associations and not put the findings into question. A possible misclassification bias might be the reason why absenteeism turned out to be the weakest of all studied work stress responses and withdrawal behaviors. In contrast, the fact that a positive and fairly strong association and a clear dose-response relationship was found between work stress and underperformance on the job suggests that there was no or not a substantial reporting bias regarding job performance.

Finally and because cross-sectional data were used, causation cannot be concluded from the identified associations. However, particularly strong associations and clear dose‒response relationships at least give an indication of a causal relationship, especially after adjustment for important control and possible confounding variables.

## Conclusion

Reducing job performance, work attendance and work engagement as well as taking unpaid leave, changing jobs, leaving the profession or at least intending to do so seem to be good “strategies” to cope with reward frustration and high levels of work stress in health care professions. In contrast, preventing such undesired withdrawal behavior from a company's or rather hospital's perspective requires prevention or at least a reduction in work stress. As expected, highly overcommitted hospital employees or health care workers tend to be even more at risk of showing such withdrawal behaviors and employees who are strongly committed to their company or employer show significantly lower risks of withdrawal behaviors. However, the two attitudes are not responsible for the strong association between work stress and withdrawal behaviors. Therefore, reward frustration in health care needs to be reduced to prevent health professionals from withdrawing from work. Since high work efforts and demands in health care mostly cannot be substantially reduced, rewards need to be increased to avoid reward frustration and reduce ERI and work stress.

Given the key role that health professionals play in health care, the follow-up costs of work stress among health professions to health care institutions, the economy and the public's health far exceed the resulting health care expenditures or mere work absences. Although these costs cannot be fully assessed, they are financially and organizationally immense.

## Data Availability

The raw data supporting the conclusions of this article will be made available by the authors, without undue reservation.
